# A need for better housing to further reduce indoor malaria transmission in areas with high bed net coverage

**DOI:** 10.1186/1756-3305-6-57

**Published:** 2013-03-07

**Authors:** Dickson W Lwetoijera, Samson S Kiware, Zawadi D Mageni, Stefan Dongus, Caroline Harris, Gregor J Devine, Silas Majambere

**Affiliations:** 1 Biomedical and Environmental Thematic Group, Ifakara Health Institute, P.O. Box 53, Ifakara, Tanzania; 2 Vector Biology Department, Liverpool School of Tropical Medicine, Pembroke Place, Liverpool, L3 5QA, UK; 3 Department of Mathematics, Statistics and Computer Science, Marquette University, Milwaukee, WI-53201-1881, USA; 4 Cairns Public Health Unit, Queensland Health, Queensland 4870, Australia

**Keywords:** House risk factors, *Anopheles gambiae s.l.*, *Anopheles funestus*, ITNs, Malaria

## Abstract

**Background:**

The suppression of indoor malaria transmission requires additional interventions that complement the use of insecticide treated nets (ITNs) and indoor residual spraying (IRS). Previous studies have examined the impact of house structure on malaria transmission in areas of low transmission. This study was conducted in a high transmission setting and presents further evidence about the association between specific house characteristics and the abundance of endophilic malaria vectors.

**Methods:**

Mosquitoes were sampled using CDC light traps from 72 randomly selected houses in two villages on a monthly basis from 2008 to 2011 in rural Southern Tanzania. Generalized linear models using Poisson distributions were used to analyze the association of house characteristics (eave gaps, wall types, roof types, number of windows, rooms and doors, window screens, house size), number of occupants and ITN usage with mean catches of malaria vectors (*An.gambiae s.l.* and *An. funestus*).

**Results:**

A total of 36490 female *An. gambiae s.l.* were collected in Namwawala village and 21266 in Idete village. As for *An. funestus* females, 2268 were collected in Namwawala and 3398 in Idete. Individually, each house factor had a statistically significant impact (*p <* 0.05) on the mean catches for *An. gambiae s.l.* but not *An. funestus*. A multivariate analysis indicated that the combined absence or presence of eaves, treated or untreated bed-nets, the number of house occupants, house size, netting over windows, and roof type were significantly related (*p <* 0.05) to *An.gambiae s.l.* and *An. funestus* house entry in both villages.

**Conclusions:**

Despite significant reductions in vector density and malaria transmission caused by high coverage of ITNs, high numbers of host-seeking malaria vectors are still found indoors due to house designs that favour mosquito entry. In addition to ITNs and IRS, significant efforts should focus on improving house design to prevent mosquito entry and eliminate indoor malaria transmission.

## Background

The *Anopheles gambiae* and *Anopheles funestus* complexes comprise the major and most efficient malaria vectors in sub-Saharan Africa [1]. Their transmission efficiency is mediated by their behavioural adaptation to feed indoors on humans [2]. To date, insecticide treated nets (ITNs) and indoor residual spraying (IRS) are the mainstay for controlling malaria vectors and associated malaria transmission [3,4]. Despite the huge success of these interventions, residual malaria transmission cannot be addressed by ITNs and IRS alone, even at very high coverage [5,6]. Moreover, their sustainability is threatened by a widespread increase in insecticide resistance in the target species [7,8]. In Senegal, the initial successes of an ITN distribution program were partially confounded by an increase in insecticide resistance and a consequent rebound in malaria incidence [9] and in northern Tanzania the predominant vector *An. arabiensis* has been reported to display avoidance behaviour against ITNs [10]. The integration of existing interventions with environmental management and socio-economic development through house improvement and screening offers a non-insecticidal, complementary approach to increasing protection against mosquito bites [11,12]. These additional interventions could enhance the interruption of malaria transmission through the reduction and prevention of human-vector contacts inside human dwellings. It has long been established that the transmission of many vector-borne diseases is facilitated by house designs that favour mosquito entry [13-15] and that housing improvements and screening have made substantial contributions to the control and elimination of malaria vectors in many richer countries [16]. Therefore, understanding house risk factors that are associated with reduction of indoor mosquito bites and disease transmission in different settings is crucial for disease vector control and elimination.

Several studies have identified and documented various house characteristics associated with mosquito entry. Presence of eave gaps, lack of a ceiling and lack of screening over windows and doors proved to be the major contributors to mosquito entry [16-20]. Furthermore, it has been shown in a randomised control trial that blocking all potential house entry points for mosquitoes substantially reduces vector densities and entomological inoculation rates (EIR) [19]. Other than protection against malaria mosquitoes, the use of screened houses offers protection against nuisance bites and other mosquito borne diseases [15,21].

While this strategy is deemed efficient in reducing indoor biting and disease morbidity in low malaria transmission settings [16], its impact is yet to be examined in areas experiencing moderate to high malaria transmission and with high ITN coverage such as the Kilombero valley in south-eastern Tanzania.

A recent study in Northern Tanzania has shown a strong association between houses, individual and behavioural risk factors and malaria transmission [22]. However, the authors argued that it was important to complement these findings with entomological data in order to have a fuller understanding of malaria transmission inside human dwellings [22]. This study therefore assessed the impact of house characteristics on indoor vector abundance in communities with a high coverage of ITNs.

## Methods

### Study site

**Figure 1:**
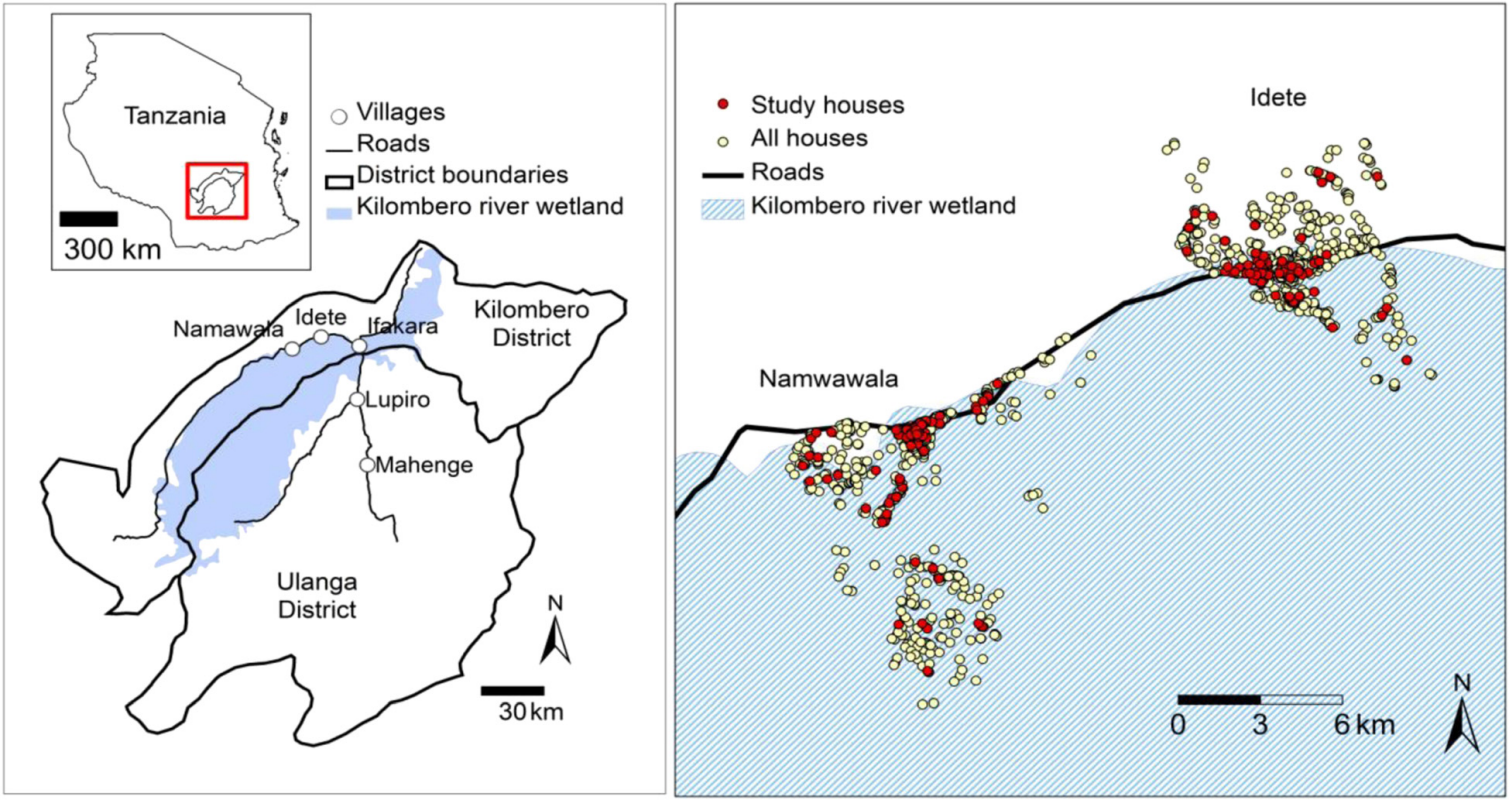
**Kilombero and Ulanga districts (8.1°S and 36.6°E) in Tanzania showing Namwawala and Idete villages (left) and spatial distribution of sentinel houses used for mosquito sampling (right) **[25]**.**

The study was carried out in Namwawala and Idete villages located in the flood plain of the Kilombero River (8.1°S and 36.6°E) in south-eastern Tanzania (Figure 1). The epidemiology of malaria transmission and associated disease vector species composition within these villages has been well studied and documented over the past years [23,24]. Both villages experience an annual rainy season (Dec – May) and the main crops are rice and maize. However, both villages have a relatively similar number of houses (Namwawala = 804 and Idete = 844), Namwawala has a high number of households (3909) compared to Idete (2932). Houses in Idete are built on relatively elevated areas compared to Namwawala. Approximately 92% of community members sleep under a treated net [23].

### Study design

This longitudinal study was conducted over four years. A total of 72 houses from each village were randomly selected from Ifakara Health Institute (IHI) Demographic Surveillance System household list [26]. All selected houses were geo-located using a handheld GPS (eTrex, Vista, Garmin, USA). Each of the 72 houses was sampled monthly (i.e. 6 houses per day, 4 days per week and 3 weeks per month). This longitudinal study was carried out between January 2008 and December 2011, during which mosquitoes were sampled every month during 2008 and 2011, for 6 months of the wet/rain season (January to June) in 2009 and for 6 months of the dry season (July to December) in 2010. This totals 36 months of sampling.

### House risk factors

**Figure 2:**
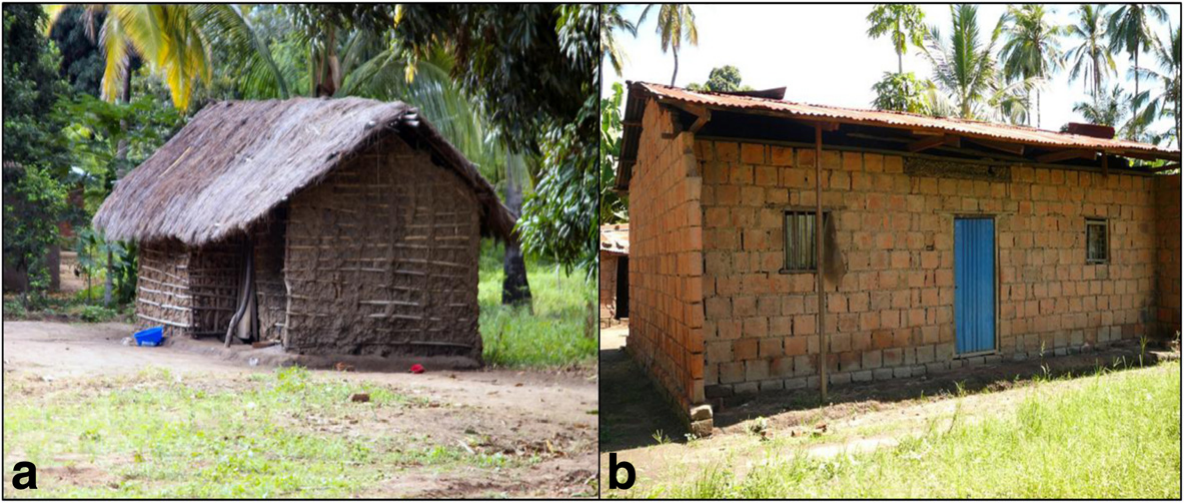
**Representative house types commonly available in Idete and Namwawala villages.** A temporary house (**a**) and a permanent house (**b**).

Structured questionnaires were used to record ownership, number and status of bed nets (either treated or untreated) including the one LLIN provided by the research team in this study, and the number of house occupants. The house characteristics which were recorded include house size, number of sleeping rooms, presence and size of eave gaps, number of windows, presence of window screening, number of doors, presence of ceiling, wall and roof types. These factors were correlated with mosquito densities indoors (an indicator of human biting rate) over time in both villages, at house level and were monitored yearly to accommodate any significant changes. Representative house types, which are commonly found in the study area are shown in Figure 2.

### Mosquito sampling and processing

Mosquitoes were sampled using miniature Centre for Disease Control (CDC) light traps (model 512, USA). One CDC light trap was set per house, placed 1–1.5 m above the ground close to the foot of a bed with an occupant sleeping under a treated net, and left to run for 12 hours (7 pm–7 am). For every participating house, one LLIN (Olyset, A to Z Textiles Mills, Arusha, Tanzania) was provided to protect the bed occupant where the CDC trap was set. Each morning of a sampling night, mosquitoes were collected and killed using chloroform and were morphologically identified in the field. Furthermore, female mosquitoes were classified as being unfed, partially fed, fully fed or gravid [2]. Sub-samples of five mosquitoes from each trap were individually stored inside a tube containing cotton wad and silica gel beneath. Polymerase chain reaction (PCR) was used for identification of *Anopheles gambiae *[27] and *An. funestus* Giles [28] complexes, whereas an enzyme-linked immunosorbent assay (ELISA) was used to determine sporozoite infection in malaria vectors [29]. Unprocessed mosquito samples were stored on silica gel at room temperature.

### Ethics

The study approval was granted by the Ifakara Health Institute Institutional Review Board (IHRDC/IRB/No.A-32) and the National Institute of Medical Research (NIMR/HQ/R.8a/Vol. IX/764). The benefits and possible risks associated with the study were explained to the house occupants before commencement. After consenting, the head of the house was asked to sign two copies of the informed consent forms, of which, one remained with the head of the house and the other copy was kept by the study investigator.

### Data analysis

The analysis was performed using generalized linear models (GLM) (MATLAB R2012a, Poisson distribution, 95% confidence interval) to assess the impact of each individual house factor on the mean catches of *An. gambiae s.l.* and *An. funestus* for both villages. Model estimate (ME) value was generated for each factor in comparison to a reference category. If the sum of ME for a factor and reference category was more than that of ME for a reference category then a factor increases indoor mean catches of mosquitoes, otherwise it decreases. Thus, ME value for a factor indicates by how much a factor increases or decreases the indoor mean catches when compared to a reference category. We categorized the house factors as follows: Eave gap: present or absent, eave gap size (small: <9 cm, medium: 9–15 cm, large > 15 cm), roof type: grass or metal roofs, wall type: mud or cement, number of occupants: up to three or more than three, windows: up to three or more than three, netting over window: intact, present but damaged or absent, doors: one or more than one, rooms: one or more than one, house size: small or large (small house considered to be the one with 1 room and/or 1 door and less than 37.4 m^3^), bed nets: treated or untreated. All houses had nets, and they were considered treated if the number of treated nets divided by the total number of nets in the house was greater than 0.5, otherwise untreated.

## Results

### Mosquito collections

A total of 36490 female *An. gambiae s.l.*, were collected in Namwawala village compared to 21266 from Idete village. Of these, approximately 98% were non-blood fed, 1.7% were blood fed and the remaining 0.3% were gravid. Namwawala had fewer female *An. funestus* 2268 than Idete village 3398. Although there were variations in catches, changes in vector abundance patterns between villages were similar over time. A PCR analysis of 6755 mosquitoes of the *Anopheles gambiae* complex yielded 607 (9%) *An.gambiae s.s.* and 6148 (91%) *An. arabiensis* mosquitoes. Furthermore, a sub-sample of 3025 *An. funestus* analyzed for species identification comprised 2805 (93%) *An. funestus s.s.*, 120 (4%) *An. rivulorum*, and 100 (3%) *An. leesoni.*

### House risk factors associated with *An. gambiae s.l.* indoor abundance

Table 1 provides parameter estimates of each house risk characteristic when run individually in a univariate model and their significance on the mean catches for *An. gambiae s.l.* All factors in both villages had a statistically significant impact (p < 0.05) on the indoor mosquito mean catches except bed net status in Namwawala (p > 0.05). Houses where an eave gap was present had significantly higher *An. gambiae s.l.* mean catches (ME 0.94 in Idete and 1.84 in Namwawala) compared to when it was absent (ME 1.50 in Idete and 1.06 in Namwawala). Mosquito density increased with more people inside the house but decreased with large houses (more rooms, windows, and doors). Compared to a window with no netting, a house with a damaged net on the window had lower mean catches of *An.gambiae s.l.* and the catches decreased further for houses with an intact net. Furthermore, houses with either mud walls or grass/thatch roofing had higher numbers of mosquitoes when compared to cement plastered walls and metal roofing.

**Table 1:** **Parameters associated with *Anopheles gambiae s.l. *density in Idete and Namwawala villages**

***An.gambiae***	**Idete (N = 70)**			**Namwawala (N = 72)**		
**Factor**	**N**	**Estimate**	**P value**	**Estimate**	**N**	**P value**
**Number Of Rooms**						
^**a**^**One**	37	2.31	0.0000	2.89	57	0.0000
**More than One**	33	-0.24	0.0035	-0.76	15	<0.0001
**Number Of Doors**						
^**a**^**One**	30	2.45	0.0000	2.93	51	0.0000
**More than One**	40	-0.47	<0.0001	-0.64	21	<0.0001
**Number Of Windows**						
^**a**^**Up to 3**	26	2.50	0.0000	2.85	53	0.0000
**More than 3**	44	-0.51	<0.0001	-0.29	19	<0.0001
**Netting Over Window**						
^**a**^**Absent**	50	2.35	0.0000	2.85	60	0.0000
**Present but damaged**	16	-0.59	0.0004	-1.65	9	<0.0001
**Intact**	4	-085	<0.0001	-0.35	3	0.0005
**House Status**						
^**a**^**Small**	12	2.76	0.0000	2.88	34	0.0000
**Large**	58	-0.71	<0.0001	-0.19	28	0.0008
**Wall Type**						
^**a**^**Mud**	52	2.65	0.0000	2.88	32	0.0000
**Cement**	18	-0.66	<0.0001	-0.28	40	<0.0001
**Roof Type**						
^**a**^**Grass**	46	2.58	0.0000	2.94	19	0.0000
**Metal**	24	-0.65	<0.0001	-0.81	53	<0.0001
**Eave Status**						
^**a**^**Absent**	46	1.50	0.0000	1.06	62	0.0000
**Present**	24	0.94	<0.0001	1.84	10	<0.0001
**Eave Size**						
^**a**^**Small**	26	1.80	0.0000	2.89	22	0.0000
**Medium**	14	0.67	<0.0001	-0.05	27	0.4452
**Large**	30	0.36	0.0028	-6.24	23	<0.0001
**Number of Occupants**						
^**a**^**Up to 3**	15	1.76	0.0000	2.35	34	0.0000
**More than 3**	55	0.54	<0.0001	0.70	38	<0.0001
**Bed-net Status**						
^**a**^**Untreated**	47	2.33	0.0000	2.58	6	0.0000
**Treated**	23	-0.45	<0.0001	0.22	66	0.0588

^a^ reference category, N = number of observations.

Note: Model estimate (ME) value for a factor indicates by how much a factor increases or decreases the indoor mean catches when compared to a reference category.

The presence of bednets was significantly correlated to lower mean catches in Idete village (p < 0.05). However, this was not the case in Namwawala village (p > 0.05). The ownership rate of nets in Namwawala village was 89% for treated and 11% for untreated nets, whereas in Idete village it was 50% for treated and 50% for untreated nets.

### House risk factors associated with *An. funestus* indoor abundance

The model estimates and *p*-values of each of the individual house risk characteristics, number of occupants and the bed-net status with their association with the mean catches for *An. funestus* for both villages are presented in Table 2. The presence of eave gap in the house was significantly correlated with increased mean catches of *An. funestus* (ME 1.42 in Idete, 2.48 in Namwawala, p < 0.05) compared to when eave gaps were absent (ME -0.73 in Idete, -2.39). House size did not significantly affect mean catches in Namwawala (p > 0.05) but in Idete mean catches for *An. funestus* decreased with large houses (ME -0.60, p < 0.05), when compared to small houses (ME 0.85). Similarly, houses with more than one room or door had lower mean catches in both villages. Increase in number of windows did not significantly affect the *An. funestus* mean catches (p > 0.05), however, the mean catches of *An. funestus* significantly decreased with increased number of people in the houses in Idete (p < 0.05) but not in Namwawala (p > 0.05). Netting over windows did not reduce the mean catches in both villages. The mean catches of *An. funestus* were significantly lower (p < 0.05) in the houses with cement plastered walls (ME -1.52 Idete, -0.55 Namwawala) compared to mud walls, as well as where metal roofs were present (ME -1.78 Idete, -0.89 Namwawala), compared to grass roofs. Mosquito catches decreased significantly (p < 0.05) in the presence of treated bednets (ME -0.52 Idete, -1.03 Namwawala) when compared to the untreated bednet (ME 0.52 Idete, ME 0.85 Namwawala).

**Table 2:** **Parameters associated with *Anopheles funestus *density in Idete and Namwawala villages**

***An.funestus***	**Idete (N = 70)**			**Namwawala (N = 72)**		
**Factor**	**N**	**Estimate**	**P value**	**Estimate**	**N**	**P value**
**Number Of Rooms**						
^**a**^**One**	37	0.76	0.0000	0.11	57	0.3485
**More than One**	33	-1.10	<0.0001	-1.25	15	0.0081
**Number Of Doors**						
^**a**^**One**	30	0.86	0.0000	0.13	51	0.3207
**More than One**	40	-1.11	<0.0001	-0.79	21	0.0167
**Number Of Windows**						
^**a**^**Up to 3**	26	0.52	0.0003	0.07	53	0.5748
**More than 3**	44	-0.26	0.1997	-0.55	19	0.0868
**Netting Over Window**						
^**a**^**Absent**	50	0.61	0.0000	0.10	60	0.3877
**Present but damaged**	16	-1.29	0.0003	-2.76	9	0.2057
**Intact**	4	-1.40	0.0603	-1.59	3	0.0251
**House Status**						
^**a**^**Small**	12	0.85	0.0000	0.12	34	0.4408
**Large**	58	-0.60	0.0065	-0.34	28	0.1534
**Wall Type**						
^**a**^**Mud**	52	1.25	0.0000	0.14	32	0.3365
**Cement**	18	-1.52	<0.0001	-0.55	40	0.0439
**Roof Type**						
^**a**^**Grass**	46	1.17	0.0000	0.13	19	0.3283
**Metal**	24	-1.78	<0.0001	-0.89	53	0.0131
**Eave Status**						
^**a**^**Absent**	46	-0.73	0.0124	-2.39	62	0.0222
**Present**	24	1.42	<0.0001	2.48	10	0.0183
**Eave Size**						
^**a**^**Small**	26	-0.42	0.0778	0.12	22	0.4612
**Medium**	14	1.04	0.0001	-0.36	27	0.1860
**Large**	30	1.21	<0.0001	-0.24	23	0.4563
**Number of Occupants**						
^**a**^**Up to 3**	15	1.05	0.0000	-0.04	34	0.8015
**More than 3**	55	-0.96	<0.0001	-0.00	38	0.9968
**Bed-net Status**						
^**a**^**Untreated**	47	0.52	0.0000	0.85	6	0.0014
**Treated**	23	-0.52	0.0273	-1.03	66	0.0005

^a^ reference category, N = number of observations.

Note: Model estimate (ME) value for a factor indicates by how much a factor increases or decreases the indoor mean catches when compared to a reference category.

### Multivariate analysis

A correlation matrix for all of the parameters was created to analyse the relationship among the house risk characteristics but no clear conclusion could be drawn. Thus, a multivariate analysis was performed using a ‘stepwise regression approach’ in which at each step the best variable (i.e. a house risk characteristic) with a significant level (p < 0.05) is added. This analysis indicated that the presence of an eave gap, bednet status, number of occupants, house size and wall type had a significant impact on the mean catches of *An.gambiae* in both Namwawala and Idete. In Namwawala, also roof type and number of doors had a significant impact on the mean catches of *An.gambiae*.

Bednet status, number of occupants, house size, roof type and number of windows had a significant impact on the mean catches of *An. funestus* in Idete while netting over windows, presence of eave gap, bednet status, and number of doors had a significant impact on the mean catches of *An. funestus* in Namwawala.

## Discussion

Despite high coverage and extensive usage of insecticide treated nets in rural communities of southern Tanzania [23], partly designed to deter and divert mosquitoes from entering houses [30], a high number of malaria vectors are still found indoors with an average of 22.22 (CI = 16.93 – 27.51) *An. gambiae s.l.* and 1.35 (CI = 1.07 – 1.63) *An. funestus* mosquitoes per trap night per house in Namwawala. In addition, an average of 13.12 (CI = 10.94 – 15.30) *An.gambiae s.l.* and 2.09 (CI = 1.56 – 2.63) An*. funestus* were collected in Idete per trap night in a house.

Small houses, constituting the majority of houses in the study area, characterized by relatively low numbers of windows, doors and rooms were associated with relatively high densities of malaria vectors. Although the association of house size and indoor mosquito density remains unknown, it was, however, assumed that smaller houses are likely to concentrate more human odours, which would attract high mosquito numbers. Conversely, houses with more sleeping rooms had a lower density of vectors because they usually have more sleeping spaces, which is likely to encourage consistent use of bed nets by sleepers [31,32]. Moreover, houses with many rooms are likely to have more nets, which collectively might reduce the number of mosquitoes indoors.

Houses made of mud walls and grass roofs had an increased risk of mosquito bites indoors. Such houses create cooler, darker conditions favoured by resting mosquitoes [33,34]. Moreover, mud walls as well as grass roofs often have crevices used by mosquitoes to enter the houses unlike cement walls and metal roofs [18]. In addition, lack of or damaged screening over windows as well as open eaves provided entry points and led to increased mosquito abundance inside the houses. These findings are consistent with other studies [16,35-38] which demonstrated that poorly constructed houses (with mud walls, grass roofs, lack of screening and with eave gaps tend to have increased human-vector exposure), resulting in a higher risk of malaria transmission.

It has been documented that houses with many occupants tend to attract vectors of disease [39,40]. In this study, the presence of many sleepers in a small house exposed them to a higher risk of *An.gambiae s.l.* bites but to a lower risk from *An. funestus.* Large amounts of human emanations from houses with more occupants tend to increase mosquito attractiveness towards that particular house compared to ones with fewer sleepers [41,42]. The observed inverse relationship between *An. funestus* and number of occupants inside the house was unexpected; however, it might be due to uneven distribution of *An. funestus* within the villages. Higher numbers of *An. funestus* collected during the dry season [43] were mostly and consistently from a cluster of a few houses located in a particular village hamlet. Therefore, the majority of houses within the sampling area experienced none or low catches.

Furthermore, significant impacts of house risk factors on *An. funestus* indoor mean catches were not consistent between villages. While this observation remains inconclusive, we postulated the cause to be exceedingly low numbers of *An. funestus* collected between villages compared to *An. gambiae s.l.*

Treated nets provided more protective advantages than untreated ones as also observed in previous studies [22,23,44,45]. However, the density of *An. gambiae s.l*. in Namwawala was higher compared to Idete despite 90% ITN coverage in Namwawala. These results indicate that even at high coverage levels, ITNs still have limitations in reducing the number of malaria vectors entering the houses. Furthermore, recent studies [46,47] have indicated that poor compliance and usage of bed nets by communities in the tropics is associated with heat discomfort associated with poor airflow caused by bed nets. Although bed nets were procured individually and there was a distribution campaign during the study period, the age of nets as well as usage of ITNs was not systematically investigated in this study, our results illustrate that a risk of transmission remains whenever people are not using treated nets in an optimal way. Improved house designs, and modifications to existing houses could substantially reduce the risk of mosquito-human contact. Although house improvement has been advocated as an efficient intervention for malaria control, the majority of houses in poor rural Africa are temporary and built with minimal material resources. This renders improvements expensive and/or impractical in most rural communities in the short term. Permanent houses (Figure 2b) could be easily and cheaply modified by screening eaves, windows and doors accompanied by community sensitization towards intervention sustainability. Temporary houses (Figure 2a) are less amenable to modifications unless they are rebuilt as more permanent structures. This would have to be addressed through a long-term strategy that sought to build better, inexpensive house models using better construction materials and sustainable financing initiatives, which can be adopted in poor settings. Such an intervention is likely to be beneficial in reducing vector borne diseases and other diseases linked to poor hygiene.

## Conclusions

This study shows the impact of specific housing characteristics on malaria vector density and the associated risk of indoor disease transmission. It also shows that even at high coverage levels of ITNs, there remains a high risk of human-mosquito contact and also that this transmission risk can be mitigated by changing house structure. Communities with permanent, spacious and screened houses are at lower risk of indoor malaria transmission.

## Competing interests

The authors have declared no competing interests.

## Authors' contributions

DWL and SM proposed the study hypothesis. DWL and SSK performed statistical analysis and wrote the first draft of the manuscript. DWL supervised the study data collections. ZDM, CH, SD & GD contributed to writing of the manuscript. All authors read and approved the final manuscript.
